# 5-Hydroxymethylfurfural Alleviates Lipopolysaccharide-Induced Depression-like Behaviors by Suppressing Hypothalamic Oxidative Stress and Regulating Neuroinflammation in Mice

**DOI:** 10.3390/antiox14111366

**Published:** 2025-11-17

**Authors:** Bailiu Ya, Haiyan Yin, Lili Yuan, Aihong Jing, Yuxuan Li, Fenglian Yan, Hui Zhang, Huabao Xiong, Mingsheng Zhao

**Affiliations:** 1 Key Laboratory of Cell and Biomedical Technology of Shandong Province, Jining Medical University, Jining 272067, China; yabailiu@mail.jnmc.edu.cn (B.Y.); yflian1117@mail.jnmc.edu.cn (F.Y.); zhanghui1024@mail.jnmc.edu.cn (H.Z.); 2 Department of Physiology, Basic Medical School , Jining Medical University, Jining 272067, China; liyuxuan@stu.mail.jnmc.edu.cn; 3 Department of Histology and Embryology, Basic Medical School , Jining Medical University, Jining 272067, China; yinhy@mail.jnmc.edu.cn (H.Y.); liliyuan06@mail.jnmc.edu.cn (L.Y.); 4 Department of Anatomy, Basic Medical School , Jining Medical University, Jining 272067, China; jingah918@mail.jnmc.edu.cn; 5 Institute of Immunology and Molecular Medicine, Jining Medical University, Jining 272067, China; 6 Jining Key Laboratory of Immunology, Jining Medical University, Jining 272067, China

**Keywords:** 5-hydroxymethyl-2-furfural, depression, oxidative stress, neuroinflammation, Nrf2

## Abstract

5-hydroxymethylfurfural (5-HMF) has been shown to exert neuroprotective effects in a global cerebral ischemia mouse model in our previous study, where it demonstrated antioxidant and anti-inflammatory properties. However, studies on its antidepressant mechanisms remain scarce. Since oxidative stress and neuroinflammation are closely associated with depression, this study investigated the antidepressant effects of 5-HMF, focusing on its potential inhibition of oxidative stress via the Nrf2 pathway and its role in microglial M1 polarization-mediated neuroinflammation. An acute depression mouse model induced by intraperitoneal injection of lipopolysaccharide (LPS) was utilized. Mice received 5-HMF (12 mg/kg) or an equal volume of vehicle via intraperitoneal injection 30 min prior to and 5 min after LPS administration. At 24 h post-modeling, behavioral tests (sucrose preference, forced swim, and open field tests) were conducted to evaluate the antidepressant effect of 5-HMF. Histological damage in the hypothalamus was assessed using Nissl staining and terminal deoxynucleotidyl transferase-mediated dUTP nick-end labeling (TUNEL) staining. Immunofluorescence was performed to evaluate M1 polarization of hypothalamic microglia. Oxidative stress damage was assessed by measuring malondialdehyde (MDA), carbonyl groups, and 8-hydroxy-2′-deoxyguanosine (8-OHdG) levels. Nrf2 DNA-binding activity was examined using an ELISA-based assay. The expression of inflammatory cytokines, Nrf2, and downstream antioxidant proteins was analyzed by ELISA kits and Western blotting. 5-HMF significantly alleviated LPS-induced depression-like behaviors, reduced hypothalamic neuronal damage, decreased oxidative stress, and inhibited microglial M1 polarization. It also regulated the expression of inflammatory cytokines (IL-1β, IL-6, TNF-α, IL-4, and IL-10) and activated the Nrf2 signaling pathway, enhancing nuclear translocation efficiency. Notably, these effects were significantly attenuated by the Nrf2 inhibitor brusatol. In conclusion, 5-HMF exerts neuroprotective effects by modulating Nrf2-mediated oxidative stress responses and suppressing microglial M1 polarization-driven neuroinflammation. These findings suggest that 5-HMF may provide therapeutic potential for alleviating depression symptoms induced by acute inflammation.

## 1. Introduction

Depression is a prevalent psychiatric condition characterized by persistent sadness, loss of interest or pleasure, and reduced vitality. It is associated with high disability, mortality, and recurrence rates [1]. Approximately 280 million people worldwide suffer from depression, accounting for 3.8% of the global population [2]. In China, more than 54 million individuals (about 4.2% of the population) are affected [3]. The molecular mechanisms of depression involve complex, interconnected pathways, including disturbances in monoamine neurotransmission, imbalances in neurotrophic support, chronic neuroinflammation, dysregulation of the hypothalamic–pituitary–adrenal axis and stress response, impaired neurogenesis, and altered excitatory-inhibitory balance [4]. Epigenetic factors, mitochondrial dysfunction, the gut–brain axis, and genetic predisposition also contribute to the diverse molecular underpinnings of this complex disease [5,6]. Among cellular and molecular mechanisms, oxidative stress and neuroinflammation play crucial roles in the onset and progression of depression.

Oxidative stress arises from an imbalance between reactive oxygen species (ROS) generation and the cellular antioxidant defense system, inducing oxidative damage to cellular lipids, proteins, and genomic DNA, which contributes to the pathogenesis and progression of numerous diseases, including major depressive disorder [7]. Studies have demonstrated that patients with depression show altered activities of antioxidant enzymes such as superoxide dismutase (SOD), whereas oxidative damage products such as malondialdehyde (MDA) are elevated relative to controls [8,9]. An animal study also showed that after induction of depression-like behaviors through stress injury, levels of brain oxidative stress markers were elevated [10]. Antioxidant defenses play a crucial role in mitigating the adverse effects of oxidative stress, and exogenous supplementation with antioxidants may be beneficial. Research indicates an association between depression and reduced intake of antioxidant vitamins (A, C, E), B vitamins, and vitamin D3 [11]. Given these findings, antioxidant therapy for depression may hold promise and could lead to pharmacological advances.

Elevated oxidative stress is recognized as a key contributor to immune dysfunction with excessive production of pro-inflammatory mediators. This amplifies neuroinflammatory responses within the central nervous system (CNS), creating a synergistic loop in which overactivated inflammatory cascades and ROS elevation jointly drive depressive pathogenesis [12]. Neuroinflammation has emerged as a central pathological mechanism in major depressive disorder (MDD), particularly involving microglia, which dynamically regulate neuroinflammatory processes through phenotypic polarization into M1 pro-inflammatory or M2 anti-inflammatory states. Dysregulated microglial polarization is now considered a critical factor in MDD etiology. Studies have demonstrated that acute stress exposure induces aberrant microglial activation and M1 polarization, triggering ROS release and secretion of pro-inflammatory cytokines, ultimately leading to neuronal damage [13,14].

5-Hydroxymethylfurfural (5-HMF) is a five-membered ring aromatic aldehyde widely present in traditional Chinese herbs, medicinal preparations, and plants with high sugar content [15]. It is also a major active component of traditional Chinese medicines such as Corni Fructus [16], Schisandra Chinensis [17] and Rehmanniae Radix [18]. In recent years, the biological activities of 5-HMF have been increasingly characterized, including antioxidant [19], anti-inflammatory [20], neuroprotective [21], and immunomodulatory effects [22]. Our previous study also demonstrated that 5-HMF exerts neuroprotective effects in mice subjected to global cerebral ischemia via bilateral common carotid artery occlusion, primarily by mitigating oxidative stress [21,23]. However, to our knowledge, the protective role of 5-HMF in depression has not yet been fully investigated, despite its potent antioxidant and anti-inflammatory properties.

5-HMF is thought to exert some of its antioxidant actions via the nuclear factor erythroid 2–related factor 2 (Nrf2) pathway [21]. Intense oxidant exposure promotes an imbalance in Nrf2 pathway. Under basal conditions, Nrf2 interacts with Kelch-like ECH-associated protein 1 (Keap1) to form a Keap1–Nrf2 complex in the cytoplasm, which suppresses Nrf2-mediated gene expression. Upon stimulation, this complex dissociates, allowing Nrf2 to translocate into the nucleus and bind to the antioxidant response element (ARE), thereby activating the transcription of ARE-dependent phase II detoxification enzymes and antioxidant defense enzymes, such as heme oxygenase-1 (HO-1). Indeed, activation of the Nrf2 pathway not only effectively inhibits oxidative stress damage but also modulates neuroinflammatory responses [24]. However, whether 5-HMF acts its antidepressant effects directly through Nrf2 remains to be confirmed.

In view of this background, we hypothesized that 5-HMF may attenuate depression-like behaviors by inhibiting oxidative stress and neuroinflammation. We employed an LPS-induced depression mouse model to investigate whether 5-HMF exerts antidepressant effects through inhibition of oxidative stress via activation of the Nrf2 pathway and suppression of microglial M1 polarization–mediated neuroinflammation in the hypothalamus.

## 2. Materials and Methods

### 2.1. Drug Treatment and Experimental Design

Male C57BL/6 mice (20–22 g) were obtained from the Pengyue Experimental Animal Breeding Institute (Jinan, China). During the experiment, the mice were maintained in a constant environment (23 ± 2 °C, 50–60% humidity, 12 h light–dark cycle) with free access to water and standard food. All procedures were performed in accordance with the ARRIVE (Animal Research: Reporting In Vivo Experiments) guidelines. The Ethics Committee of Jining Medical University approved all experimental protocols (No. JNMC-2020-DW-012). Every effort was made to minimize animal suffering and reduce the number of animals used.

After a one-week adaptation period, mice were randomly divided into four groups: Control, LPS + vehicle, LPS + 5-HMF (12 mg/kg), and LPS + 5-HMF (12 mg/kg) + Brusatol (1.0 mg/kg). Mice were injected intraperitoneally with either LPS (1 mg/kg) or 0.9% saline, as previously described [25,26,27]. 5-HMF (12 mg/kg) or an equal volume of vehicle was administered via tail vein injection 30 min prior to and 5 min after LPS administration. In the LPS + 5-HMF + Brusatol group, the Nrf2 inhibitor brusatol (1 mg/kg, dissolved in 1% DMSO) was administered intraperitoneally 1 h prior to modeling. The other three groups received an equal volume of vehicle. Water was deprived for 24 h before LPS injection, after which the sucrose preference test (SPT) was conducted within 0–24 h post-LPS injection, followed by the forced swimming test (FST) and the open field test (OFT). Animals were euthanized 24 h after LPS administration, and brains were collected for histological examination, Western blotting, OxyBlot analysis, and biochemical assays.

### 2.2. Behavioral Assessments

#### 2.2.1. Sucrose Preference Test (SPT)

During the initial 24 h phase, mice were housed individually with two bottles containing a 2% sucrose solution. In the subsequent 24 h phase, one bottle was replaced with sterilized water, and the positions of the bottles were alternated every 12 h to prevent position bias. Following adaptation, all mice underwent 24 h of food and water deprivation. Thereafter, they received drug administration according to the experimental design. Each cage was then provided with equal volumes of 2% sucrose solution and water for the formal test. The volumes of sucrose solution and water consumed were measured to determine liquid intake. Sucrose preference was calculated as: Sucrose preference (%) = 100% × sucrose consumption/(sucrose consumption + water consumption).

#### 2.2.2. Forced Swim Test (FST)

The experimental protocol for the forced swim test was as follows: Twenty-four hours prior to testing, animals were housed in a standardized room (illumination < 50 lux) for acclimation. The testing apparatus consisted of transparent acrylic cylindrical containers (diameter = 10 cm, height = 30 cm) filled with water maintained at 24 ± 1 °C to a depth of 25 cm. The formal testing duration was 6 min, with the initial minute designated for acclimation and the subsequent 5 min recorded using a video tracking system (CleverSys Inc., Fairfax, VA, USA). Immobility time—defined as the duration during which the animal exhibited only minimal movements necessary to remain afloat—was the primary outcome measure.

#### 2.2.3. Open Field Test (OFT)

The experimental apparatus consisted of a square open box (100 cm × 100 cm × 50 cm) with a camera system mounted above. Before testing, the arena floor was digitally divided into nine equal squares using analysis software. Mice were placed in the central zone at the beginning of each trial, and their exploratory behavior was recorded for 5 min with a video tracking system (ANY-maze, Stoelting Co., Wood Dale, IL, USA). Rearing frequency, total distance traveled, and entries into the central zone were analyzed with ANY-maze software (version 7.49).

### 2.3. Immunofluorescence and Nissl Staining

Mice were transcardially perfused with PBS followed by 4% paraformaldehyde (*n* = 4 per group). Coronal brain sections from bregma −1.50 to −2.00 mm were cut at a thickness of 25 μm. Sections were grouped into four sets: the first set was used for Nissl staining, the second for terminal deoxynucleotidyl transferase-mediated dUTP nick-end labeling (TUNEL) staining, the third for 8-hydroxy-2′-deoxyguanosine (8-OHdG) staining, and the fourth for immunofluorescence staining. Sections were incubated with primary antibodies: anti-mouse Iba-1 IgG mAb (1:500, OB-MMS039, Oasis biofarm, Hangzhou, Zhejing, China ), anti-rabbit CD86 antibody (1:200, CY5238, Abways, Shanghai, China), and anti-8-OHdG antibody (1:100, sc-393871, Santa Cruz Biotechnology, Texas, USA). After primary incubation, sections were incubated with AF-488– or AF-594–conjugated secondary antibodies.

Nissl staining was performed according to standard procedures. The TUNEL detection kit (C1089; Beyotime Biotechnology, Shanghai, China) was used to measure DNA fragmentation. Sections were counterstained with 4′,6-diamidino-2-phenylindole. Negative controls underwent the same staining procedures except for omission of the primary antibody. Fluorescence signals were detected with a fluorescence microscope (Nikon, Tokyo, Japan), and the numbers of Iba-1^+^, CD86^+^Iba-1^+^, Nissl^+^, TUNEL^+^, and 8-OHdG^+^ cells in hypothalamic sections from each animal were analyzed using ImageJ software (version 1.54g).

### 2.4. Network Pharmacological Analysis

Potential target candidates for 5-HMF were collected from SwissTargetPrediction (http://www.swisstargetprediction.ch/, accessed on 15 July 2023) and PharmMapper (https://www.lilab-ecust.cn/pharmmapper/ , accessed on 15 July 2023). Gene symbols of candidate targets were annotated using the UniProt database (https://www.uniprot.org/, accessed on 30 July 2023). Depression-related targets were obtained from the Online Mendelian Inheritance in Man (OMIM, https://omim.org/, downloaded 20 July 2023) [28] and GeneCards (version 5.17, updated 2 August 2023, https://www.genecards.org/) databases [29], whereas oxidative stress– and neuroinflammation-related targets were obtained from GeneCards using “oxidative stress, neuroinflammation, depression” as the keywords. The intersection of these four databases was used to identify expression targets associated with oxidative stress and neuroinflammation. Shared genes between drug targets and disease targets were analyzed and visualized using the Bioinformatics platform (www.bioinformatics.com.cn, accessed on 26 August 2023). Gene Ontology (GO) enrichment and Kyoto Encyclopedia of Genes and Genomes (KEGG) pathway analysis of the disease–compound intersection genes were performed using Metascape (https://metascape.org/, accessed on 28 August 2023 ). The protein–protein interaction (PPI) network for all 5-HMF targets in depression associated with oxidative stress and neuroinflammation was generated using STRING (https://cn.string-db.org/, accessed on 30 August 2023, confidence score ≥ 0.4) and visualized with Cytoscape software (Cytoscape_v3.8.2).

### 2.5. Western Blot

The hypothalamus (n *n* = 4 per group) was dissected, and total protein was extracted using a total protein extraction kit (Applygen Technologies Inc., Beijing, China). Nuclear and cytoplasmic fractions were isolated using a nuclear–cytosol extraction kit (Applygen Technologies Inc., Beijing, China). Cytoplasmic protein was used to examine HO-1 and Nrf2, whereas nuclear protein was collected for Nrf2 analysis. Protein loading was standardized at 50 μg per sample. The primary antibodies used were HO-1 polyclonal antibody (10701-1-AP, Proteintech Group, Wuhan, China) and Nrf2 polyclonal antibody (CY5136, Abways, Shanghai, China). For analysis, target protein levels were normalized to histone H3 or β-actin, and results were expressed as intensity ratios relative to control (% of control).

### 2.6. Biochemical Analyses

The supernatant of the hypothalamus was collected for the detection of SOD, catalase (CAT), and glutathione (GSH). All biochemical parameters were measured using commercial kits (Nanjing Jiancheng Bioengineering Institute, Nanjing, China). The concentrations of IL-1β, IL-6, TNF-α, IL-4, and IL-10 in the hypothalamus were determined using ELISA kits (Boster Biological Technology Co., Ltd., Wuhan, China) according to the manufacturer’s instructions. Optical density was measured with a spectrophotometer at 450 nm.

Nrf2 DNA-binding activity was determined using the TransAM^®^ Nrf2 ELISA kit (Active Motif, Carlsbad, CA, USA) following the manufacturer’s protocol. Nuclear protein (10 μg) was incubated with a DNA probe containing the Nrf2-binding sequence at room temperature for 1 h to allow Nrf2 in the nuclear extract to bind to the DNA probe. The mixture was then transferred to react with an HRP-conjugated antibody, forming HRP-labeled DNA–Nrf2 complexes. The signal was measured using a microplate reader at 450 nm. The intensity of the signal was proportional to the amount of Nrf2 bound to the DNA probe, reflecting Nrf2 DNA-binding activity.

### 2.7. Determination of Protein Oxidation and Lipid Peroxidation

To assess protein oxidation, the OxyBlot™ Protein Oxidation Detection Kit (#S7150; Millipore, MA, USA) was used as previously described. Protein carbonyl content, a biomarker of oxidative damage, was quantified with this commercial kit. Cytoplasmic supernatants derived from tissue samples were incubated with 2,4-dinitrophenylhydrazine (DNP) to derivatize carbonyl groups. Subsequent Western blot analysis employed anti-DNP antibodies to specifically detect modified proteins, and signal intensities were scanned and quantified.

MDA, one of the most widely used markers of lipid peroxidation, was measured using the MDA Detection Kit (Nanjing Jiancheng Bioengineering Institute, Nanjing, China) according to the manufacturer’s instructions. MDA reacts with thiobarbituric acid under acidic and high-temperature conditions to form a pink chromogen with absorption at 532 nm. The intensity of the color was proportional to the amount of MDA present in the sample.

### 2.8. Statistical Analysis

All data in this study were expressed as mean ± standard error (SE). Statistical analyses were performed using one-way analysis of variance. LSD test and Student-Newman–Keuls test were used as the post hoc analysis. Differences with *p* < 0.05 were considered statistically significant.

## 3. Results

### 3.1. 5-HMF Reversed LPS-Induced Depression-like Behaviors in Mice

**Figure 1:**
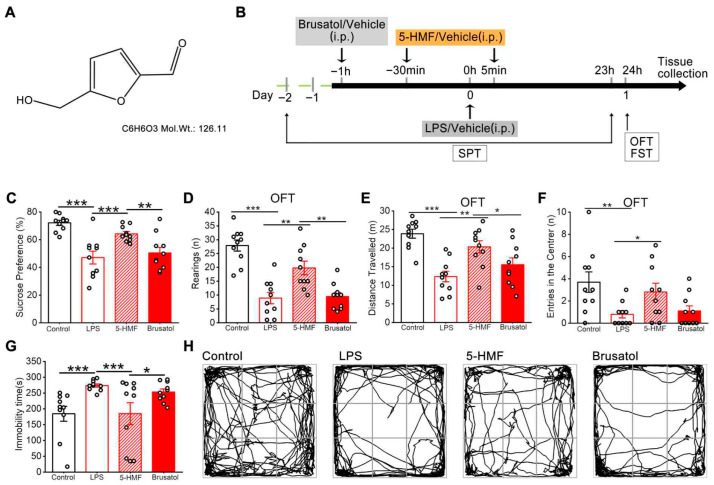
5-HMF improved depression-like behavior in LPS-induced mice. (**A**) Chemical structure of 5-HMF. (**B**) Schematic representation of the experimental procedure. (**C**) Sucrose preference in the SPT. (**D**) Rearing frequency in the OFT. (**E**) Total distance traveled in the OFT. (**F**) Entries into the central zone in the OFT. (**G**) Immobility time in the FST. (**H**) Representative movement trajectory in the OFT. LPS, lipopolysaccharide; SPT, sucrose preference test; OFT, open field test; FST, forced swim test. Results are expressed as mean ± SEM, *n* = 10 per group.* *p *< 0.05, ** *p* < 0.01, *** *p* < 0.001.

Figure 1A and 1B show the chemical structure of 5-HMF and the experimental scheme, respectively. The antidepressant efficacy of 5-HMF against LPS-induced depression-like behaviors in mice was systematically evaluated using the SPT, OFT, and FST. As shown in Figure 1C, LPS administration significantly reduced sucrose preference, a validated marker of anhedonia, whereas 5-HMF treatment reversed this effect. In the OFT, LPS-exposed mice exhibited pronounced reductions in rearing frequency, total distance traveled, and central zone entries. Notably, 5-HMF administration restored these deficits in spatial exploration, as evidenced by increased central zone occupancy and movement trajectories in trace map analysis (Figure 1D–F,H). In the FST, LPS markedly prolonged immobility time, a behavioral indicator of despair, which was significantly attenuated following 5-HMF treatment (Figure 1G). Brusatol suppressed the antidepressant efficacy of 5-HMF, as demonstrated by decreased sucrose preference and exploratory behavior, together with prolonged immobility in the FST. Collectively, these findings demonstrate that 5-HMF exerts robust antidepressant effects by mitigating core depression-related phenotypes, including anhedonia, behavioral despair, and reduced exploratory activity, in LPS-challenged mice.

### 3.2. Network Pharmacology Analysis of 5-HMF in Depression Associated with Oxidative Stress and Neuroinflammation

**Figure 2:**
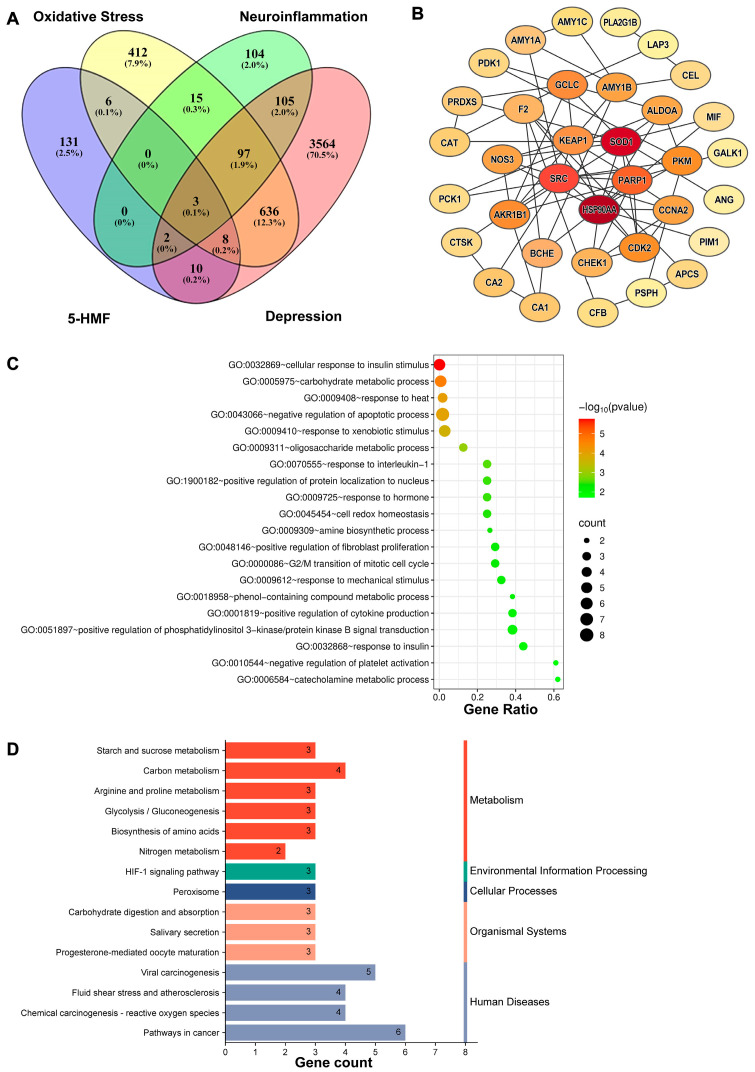
Network pharmacological analysis of 5-HMF in depression associated with oxidative stress and neuroinflammation. (**A**) Venn diagram of 5-HMF target genes and disease-related target genes. The pink oval represents depression target genes, the purple oval represents effective 5-HMF target genes, the yellow oval represents oxidative stress target genes, and the green oval represents neuroinflammation target genes. (**B**) PPI network of the target genes shared between 5-HMF and depression-associated oxidative stress and neuroinflammation. The darker the color of the nodes, the more important the genes represent. (**C**) Gene Ontology (GO) biological process enrichment of the shared candidate targets. (**D**) Kyoto Encyclopedia of Genes and Genomes (KEGG) pathway enrichment of the shared candidate targets.

SwissTargetPrediction and PharmMapper analyses identified 160 potential targets of 5-HMF. Screening revealed 744 depression-associated oxidative stress targets and 207 depression-associated neuroinflammation targets. A Venn diagram showed 11 potential targets of 5-HMF in oxidative stress associated with depression and 5 potential targets in neuroinflammation associated with depression (Figure 2A). Enrichment analysis of GO biological process terms revealed significant associations with the negative regulation of apoptotic processes, cell redox homeostasis, and positive regulation of cytokine production (Figure 2C). KEGG pathway enrichment analysis clustered the major effects of 5-HMF in depression associated with oxidative stress and neuroinflammation. A total of 15 top-ranking pathways were identified. The main pathways included the HIF signaling pathway, pathways in cancer, and pathways related to metabolism (Figure 2D). The protein–protein interaction (PPI) network contained 35 nodes, among which SOD1, HSP90AA1, SRC, PARP1, and KEAP1 were considered hub genes (Figure 2B). These findings suggest that 5-HMF exerts antidepressant effects through the modulation of oxidative stress and neuroinflammation. Structurally, 5-HMF contains an α,β-unsaturated carbonyl group, enabling direct interaction with sulfhydryl groups of cysteine residues in Keap1 as an Nrf2 inducer. This interaction promotes the dissociation of Nrf2 from the Keap1–Nrf2 complex, facilitating its nuclear translocation and subsequent transcription of antioxidant genes. The regulatory role of 5-HMF as an Nrf2 inducer acting on the Keap1–Nrf2 complex in depression will be confirmed in this study.

### 3.3. 5-HMF Attenuated Neuronal Injury in the Hypothalamic Region of LPS-Induced Mice

**Figure 3:**
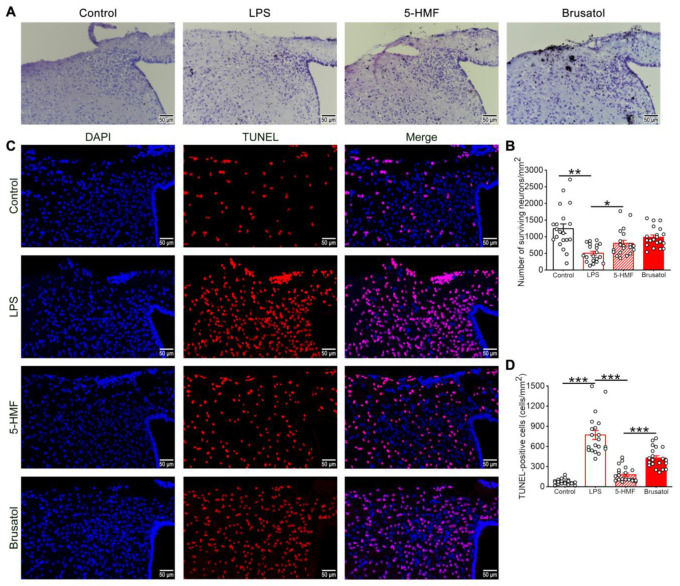
5-HMF ameliorated neuronal damage in the hypothalamus of LPS-induced mice. (**A**) Representative photomicrographs of Nissl staining in the hypothalamus; scale bar, 50 μm; *n* = 4 per group. (**B**) Quantitative analysis of Nissl-positive cells. (**C**) Representative photomicrographs of TUNEL staining in the hypothalamus. (**D**) Quantitative analysis of TUNEL-positive cells; scale bar, 50 μm; *n* = 4 per group. Results are expressed as mean ± SEM. * *p* < 0.05, ** *p* < 0.01, *** *p* < 0.001.

Nissl staining was performed to evaluate the neuroprotective effects of 5-HMF against LPS-induced hypothalamic neuronal injury. As shown in Figure 3A,B, LPS administration caused marked neuronal degeneration, characterized by significant reductions in Nissl-positive cell counts and disrupted cellular morphology and arrangement. These pathological changes were attenuated following 5-HMF treatment. Notably, co-administration of brusatol significantly reduced the neuroprotective effects of 5-HMF, as evidenced by decreased Nissl staining intensity.

The protective effect of 5-HMF against LPS-induced hypothalamic neuronal injury was further confirmed by TUNEL staining (Figure 3C,D). Compared with controls, which showed minimal TUNEL-positive cells, LPS exposure significantly increased apoptosis. Treatment with 5-HMF reduced the number of TUNEL-positive cells compared with the LPS group. However, brusatol co-administration (5-HMF + Bru) reversed this effect, increasing apoptosis and indicating that brusatol attenuates 5-HMF-mediated neuroprotection. These findings demonstrate that 5-HMF exerts its neuroprotective functions, which is critical for mitigating LPS-induced hypothalamic neurotoxicity.

### 3.4. 5-HMF Alleviated Oxidative Stress in the Hypothalamic Region of LPS-Induced Mice

**Figure 4:**
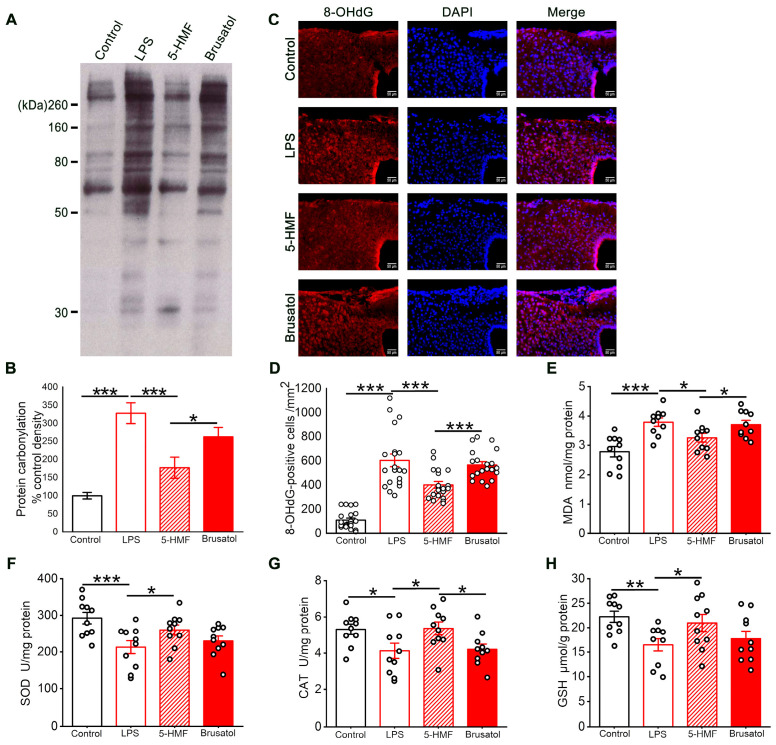
5-HMF mitigated oxidative stress in the hypothalamus of LPS-induced mice. (**A**) Representative blot of protein oxidation. (**B**) Representative photomicrographs of 8-OHdG immunostaining; scale bar, 50 μm. (**C**) Quantitative evaluation of the optical density of protein carbonyl bands; results are presented as mean ± SE, *n* = 4 per group. (**D**) Quantification of 8-OHdG-positive cells, *n* = 4 per group. (**E**) Quantification of MDA levels in hypothalamic homogenates, *n* = 10 per group. (**F**–**H**) Quantification of SOD, CAT, and GSH levels in hypothalamic homogenates, *n* = 10 per group. Data are expressed as mean ± SE. * *p* < 0.05, ** *p* < 0.01, *** *p* < 0.001.

Oxidative stress levels in the hypothalamus of the different treatment groups were assessed. LPS exposure markedly elevated concentrations of protein carbonyls, 8-OHdG, and MDA, indicating oxidative damage to proteins, DNA, and lipids. Treatment with 5-HMF significantly reduced these biomarkers, underscoring its antioxidant potential. Notably, brusatol pretreatment counteracted 5-HMF-mediated protection, as evidenced by increased protein carbonyl, 8-OHdG, and MDA levels in hypothalamic tissue (Figure 4A–E). Further analysis revealed that LPS administration significantly decreased SOD, CAT, and GSH levels in hypothalamic tissue (Figure 4F–H), indicating compromised antioxidant defenses. These perturbations were markedly reversed by 5-HMF treatment, suggesting its capacity to restore redox homeostasis.

### 3.5. 5-HMF Activated the Nrf2 Pathway in the Hypothalamic Region of LPS-Induced Mice

**Figure 5:**
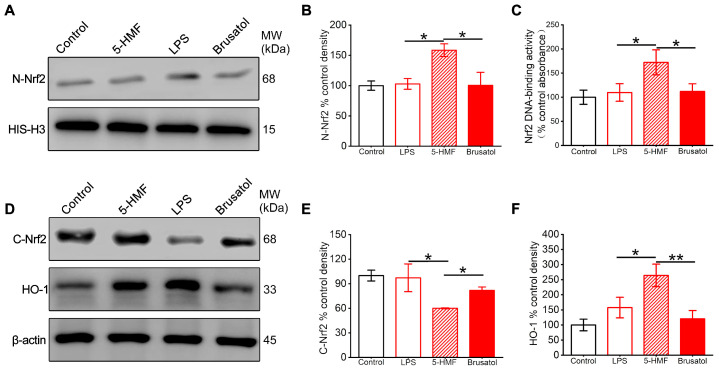
5-HMF activated the Nrf2 pathway in the hypothalamus of LPS-induced mice. (**A**) Representative Western blot of nuclear Nrf2. (**B**) Quantification of nuclear Nrf2 normalized to histone H3. (**C**) Nuclear Nrf2 DNA-binding activity. (**D**) Representative Western blot of cytosolic Nrf2 and HO-1. (**E**,**F**) Quantification of cytosolic Nrf2 and HO-1 normalized to β-actin. Data are expressed as mean ± SEM. * *p* < 0.05, ** *p* < 0.01.

The expression levels of proteins related to the Nrf2 pathway in the hypothalamic region of different treatment groups, in the presence of the Nrf2 inhibitor brusatol, were assessed. 5-HMF promoted nuclear translocation of Nrf2, with nuclear protein levels increasing 1.6-fold compared with controls (Figure 5A,B). Concurrently, cytosolic Nrf2 decreased by 40%, indicating enhanced transcriptional activation. Brusatol pretreatment significantly impaired this nuclear translocation, reducing nuclear Nrf2 accumulation by 58% (Figure 5A,B,D,E). Downstream analysis demonstrated 5-HMF–induced upregulation of HO-1 by 2.6-fold, an effect abolished by Nrf2 inhibition (Figure 5D,F). Electrophoretic mobility shift assay results confirmed enhanced Nrf2 DNA-binding activity in 5-HMF–treated animals, which was reduced by 35% following brusatol co-treatment (Figure 5C).

### 3.6. 5-HMF Suppressed Microglial M1 Polarization and Regulated Inflammatory Cytokine Levels in the Hypothalamic Region of LPS-Induced Mice

**Figure 6:**
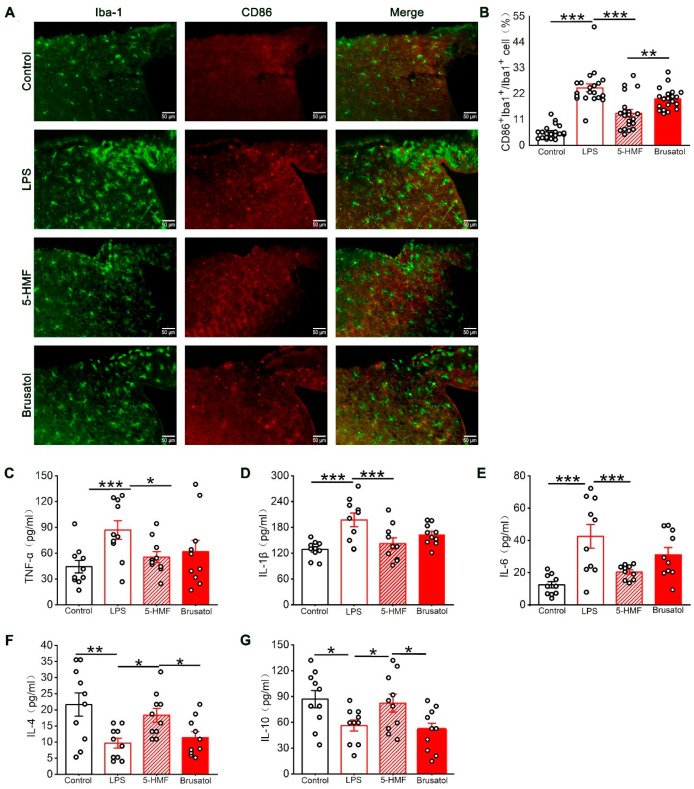
5-HMF reduced microglial M1 polarization and regulated inflammatory cytokine levels in the hypothalamus of LPS-induced mice. (**A**) Representative immunofluorescence images of the hypothalamic region co-stained with Iba-1 (green, microglial marker) and CD86 (red, M1-polarized microglial marker). Scale bar, 50 μm. (**B**) Quantification of the percentage of M1-polarized microglia among total microglia in the hypothalamus, *n* = 4 per group. (**C**–**G**) Quantification of TNF-α, IL-1β, IL-6, IL-4, and IL-10 expression levels, *n* = 10 per group. Data are expressed as mean ± SEM. * *p* < 0.05, ** *p* < 0.01, *** *p* < 0.001.

To further elucidate the effects of 5-HMF on LPS-induced neuroinflammation, microglial M1 polarization in the hypothalamus was examined by double immunofluorescence staining using the pan-microglial marker Iba-1 and the M1 polarization–associated marker CD86. Quantitative analysis revealed that LPS administration significantly enhanced CD86/Iba-1 colocalization compared with control animals. This M1 polarization was substantially attenuated by 5-HMF pretreatment, demonstrating its inhibitory effect on microglial pro-inflammatory activation (Figure 6A,B). Next, the effects of 5-HMF on inflammatory cytokines in the hypothalamus of LPS-challenged mice were examined by ELISA. LPS exposure induced a pronounced elevation of pro-inflammatory cytokines (TNF-α, IL-1β, and IL-6) alongside suppression of anti-inflammatory mediators (IL-4 and IL-10). Treatment with 5-HMF effectively normalized these alterations, reducing pro-inflammatory cytokine levels by 28–53% and increasing anti-inflammatory cytokines by 1.5–1.8-fold compared with the LPS-treated group (Figure 6C–G). These findings indicate that 5-HMF ameliorated neuroinflammation in LPS-challenged mice by suppressing microglial M1 polarization and modulating inflammatory cytokine levels in the hypothalamus. Compared with the 5-HMF group, brusatol co-administration reversed these protective effects, increasing M1 polarization frequency by 46% and exacerbating cytokine dysregulation.

## 4. Discussion

This study investigated whether 5-HMF ameliorates systemic inflammation–induced depression-like behavior in LPS-treated mice. Behavioral evaluations, including the SPT, OFT, and FST, demonstrated that 5-HMF alleviated depression-like behaviors in LPS-challenged mice. Oxidative stress assays revealed that 5-HMF enhanced hypothalamic antioxidant capacity, thereby attenuating oxidative damage. Histopathological observations indicated reduced neuronal injury following 5-HMF administration. Network pharmacological analyses highlighted Nrf2 as a key regulatory hub, whose activation potentiated the antidepressant and antioxidant effects of 5-HMF and modulated neuroinflammation. Consistently, biochemical and immunofluorescence analyses confirmed that 5-HMF suppressed microglial M1 polarization and regulated hypothalamic inflammatory cytokine levels in LPS-treated mice.

Experimental LPS is widely used to establish rodent models of neuroinflammation that reliably induce depression-like behavior following systemic administration [30]. Depression can arise in endotoxic shock due to a systemic inflammatory response mediated by cytokines such as TNF-α and IL-6. These cytokines impair brain function by disrupting the blood–brain barrier (BBB) and altering neurotransmitter systems. Furthermore, endotoxin exposure directly induces depressive symptoms in both humans and animals, such as anhedonia and sleep disturbances. This evidence suggests a direct link between the inflammatory cascade and mood changes [31,32]. Nevertheless, sickness behaviors induced by LPS administration are not consistently separable from depressive-like phenotypes [33]. Notably, most studies on LPS-induced depressive-like behaviors evaluate behavioral changes at a 24 h time-point after administration of endotoxin, as evidence indicates that depression-like behaviors can be dissociated from sickness 24 h after LPS exposure [34,35,36]. Substantial evidence has shown that LPS-treated mice exhibit heightened oxidative stress in brain tissue, characterized by significant oxidative damage and impaired antioxidant systems [37,38]. Brain regions involved in stress responses, particularly the hypothalamic–pituitary–adrenal (HPA) axis, are highly vulnerable to inflammatory challenges [39]. The hypothalamus, an integral component of the HPA axis, regulates stress responses through this primary endocrine pathway and is strongly implicated in depressive pathogenesis [40]. Thus, activation of neuroinflammatory responses and disturbances in oxidative stress within the hypothalamus represent pivotal factors contributing to the development of depression-like behaviors.

Although the neuroprotective effects of 5-HMF mediated by its antioxidative properties have been well documented, its antidepressant activity and underlying mechanisms have not yet been reported. Accumulating evidence highlights the antidepressant potential of targeting oxidative stress in preclinical depression models, establishing redox regulation as a viable therapeutic strategy for depression [41]. 5-HMF has previously demonstrated robust antioxidant properties, and the present study extends these findings by elucidating its antidepressant mechanism through oxidative stress modulation in LPS-treated mice. LPS administration significantly reduced SOD, CAT, and GSH levels in the hypothalamus, concurrent with marked elevations in oxidative stress markers, including MDA, 8-OHdG, and protein carbonyls. Notably, 5-HMF treatment reversed these pathological changes, underscoring oxidative stress as a central mediator of its antidepressant effects. Furthermore, 5-HMF increased Nissl-positive neuronal counts and reduced TUNEL-positive apoptotic cells, confirming its neuroprotective role in mitigating depression-related neuropathology.

Previous studies have indicated that neuroinflammation, a well-established driver of neuronal injury, is increasingly recognized as a critical pathogenic mechanism in depressive disorders [42]. Our findings demonstrated that 5-HMF treatment significantly regulated inflammatory cytokine levels in the hypothalamus of LPS-challenged mice. Increasing evidence highlights that M1-polarized microglia act as key regulators of neuroinflammatory processes [43]. In the present study, LPS exposure markedly enhanced hypothalamic microglial M1 polarization, an effect significantly attenuated by 5-HMF administration. These results suggest that inhibition of microglia-mediated neuroinflammation contributes to the antidepressant actions of 5-HMF.

The Nrf2 inhibitor brusatol effectively reversed the behavioral deficits induced by the LPS challenge. Notably, co-administration of brusatol with 5-HMF led to a significant increase in the number of TUNEL-positive cells compared to the 5-HMF alone group. Furthermore, pretreatment with brusatol abrogated the neuroprotective effects mediated by 5-HMF, as evidenced by elevated levels of protein carbonyls, 8-OHdG, and MDA; enhanced M1 microglial polarization; and reduced IL-4 and IL-10 production in hypothalamic tissues. These findings collectively indicate that Nrf2 serves as a critical regulatory factor underlying the protective actions of 5-HMF in LPS-challenged mice. However, brusatol failed to alter the levels of SOD and GSH, as well as the expression of TNF-α, IL-1β, and IL-6. This observation suggests that the regulatory mechanism of 5-HMF may be influenced by additional co-factors or alternative signaling pathways that remain unexplored in the present study. Beyond Nrf2, accumulating evidence from previous studies has implicated the apurinic/apyrimidinic endonuclease/redox factor-1 (APE/Ref-1) and N-methyl-D-aspartate (NMDA) receptor signaling pathways in the neuroprotective roles of 5-HMF [19,44]. Therefore, future investigations should focus on exploring the potential crosstalk between 5-HMF and these diverse signaling pathways to fully elucidate its mechanisms.

Emerging evidence shows that high doses of 5-HMF (>75 mg/kg) can be cytotoxic to cells and tissues, leading to increased oxidative stress markers and subsequently impairing cellular defense and survival mechanisms, thereby contributing to the onset and progression of various diseases [45,46,47,48]. However, several studies have indicated that 5-HMF may act as a hormetic agent, following a U-shaped dose–response curve: low-dose effects stimulate neuronal adaptive responses, while high-dose effects promote detrimental outcomes. This biphasic pattern shows consistency with the concept of hormesis—a novel, biphasic dose–response process by which small, nontoxic, or mild stresses induce cellular adaptive responses that protect biological systems against subsequent large and potentially lethal stresses of the same, similar, or different nature [49,50].

Despite the current findings, this study has several limitations. First, while the LPS-induced mouse model simulates inflammation-driven depressive-like behaviors, it may not fully replicate the complexity of human depression, given its multifaceted etiology involving neurochemical, neuroendocrine, and neuroimmune dysregulation. Further exploration and validation using other animal models should be addressed in future study. Second, the role of the Nrf2 signaling pathway might be modulated by unexplored pathways or co-factors. A broader molecular framework investigating multi-target effects of 5-HMF is needed to elucidate its mechanism. Third, it is better to strengthen evidence to validate the function of Nrf2 using genetic deletion or other Nrf2 inhibitors.

## 5. Conclusions

**Figure 7:**
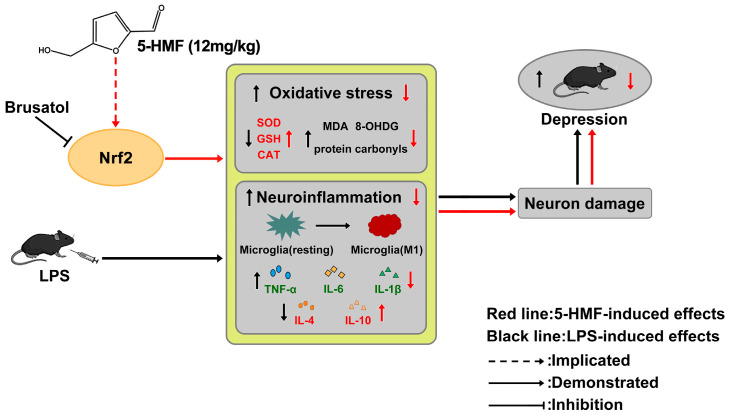
Schematic diagram depicting the antidepressant effect of 5-HMF through inhibition of oxidative stress and neuroinflammation. In LPS-treated mice, increased oxidative stress and neuroinflammation lead to neuronal damage, thereby mediating depression-like behaviors. 5-HMF enhanced antioxidant capacity and ameliorated neuroinflammation partly through activation of the Nrf2 pathway, producing antidepressant effects. The protective action of 5-HMF was abolished by the Nrf2 inhibitor brusatol.

Our study demonstrated that 5-HMF enhances hypothalamic antioxidant capacity partially through Nrf2 signaling and mitigates neuroinflammation in mice subjected to systemic LPS challenge. These effects protected hypothalamic neurons and reversed LPS-induced depression-like behavior. Notably, the neuroprotective actions of 5-HMF were partially abolished by the Nrf2 inhibitor brusatol. Collectively, these findings expand the potential preventive and therapeutic applications of 5-HMF for depression and highlight a promising avenue for future translational research into novel antidepressant strategies (Figure 7).

## Data Availability

The original contributions presented in the study are included in the article, further inquiries can be directed to the corresponding authors.
